# Editorial: Training readers and writers for a multimodal and multimedia society: cognitive aspects

**DOI:** 10.3389/fpsyg.2024.1357773

**Published:** 2024-01-30

**Authors:** Ester Trigo Ibáñez, Inmaculada Clotilde Santos Díaz, Marcela Jarpa Azagra

**Affiliations:** ^1^Department of Language Teaching, University of Cádiz, Cádiz, Spain; ^2^Department of Didactics of Languages, Arts and Sports, University of Málaga, Málaga, Spain; ^3^Faculty of Philosophy and Education, Pontificia Universidad Católica de Valparaíso, Valparaíso, Chile

**Keywords:** cognition, critical thinking, lexical availability, reading habits, academic writing, writing in L2, literacy

This Research Topic includes 10 articles belonging to the educational, psychological, and linguistic fields with researchers from different universities in Chile, China, and Spain. The articles have been published in the “Psychology of Language,” “Educational Psychology,” and “Cognition” sections of the journal Frontiers in Psychology, and the “Language, Culture, and Diversity” section of the journal Frontiers in Education.

Modern societies have made steady progress in creating a literate population. The majority of people can now access a wide range of resources using their reading and writing skills thanks to significant efforts, but are we really ready for the information age? Are educational institutions promoting information literacy? Are personal skills being considered? Are we really critical thinkers? This Research Topic provides an overview of the latest studies, covering issues from the individual to the group, from the skills of the subject to how best to develop them in the classroom.

Our main objective was to address a pressing issue facing modern global societies. It was suggested that the Research Topic explore this by providing a range of insights into critical literacy in general and reading and writing in particular. Research on writing processes, academic literacy, and analog and digital reading was therefore invited. This Research Topic also considered the cognitive processes of the subject in relation to building their mental lexicon and accessing information. This provides a glimpse into the world of people living in the post-truth era and immersed in literacy.

The articles published in this Research Topic have examined critical literacy from a variety of perspectives, providing a thorough examination of the contemporary heterogeneous cultural, linguistic, educational, and psychological environment, as outlined below. They are divided into four sections: reading, writing, foreign language teaching, and cognition.

In the first section, there are two studies that focus on the writing process. To investigate the effects of working memory updating training on primary school students' writing performance and ability, Gao, Li et al. conducted a study entitled “*The effect of working memory updating training on the Chinese writing ability of primary school students*.” Sologuren's research, on the other hand, describes the rhetorical-discursive organization of the laboratory report genre in this subdiscipline. It is entitled “*Student writing in the engineering curriculum: discursive rhetorical model of the laboratory report genre in Spanish*”.

In the second section, three articles explore the cognitive and cultural dimensions of learning. Using conceptual metaphors as the guiding theory, Li and Lu's study, “*Conceptual metaphors and image construction of China in the space probe reports of China Daily: a social cognitive approach*,” examines conceptual metaphors in China Daily news releases about Chang'e-5 and Shenzhou XIII from 2008 to 2021. The goal of de la Montaña Conchiña et al.'s research, “*Conceptions of geography and history as school disciplines: an approach from lexical availability*,” aims to investigate how students at different educational levels perceive geography and history as academic subjects. Finally, Muñoz-Muñoz et al.'s paper, “*The influence of transmedia and extra-academic narratives on the formation of high school students' historical culture*,” focuses on the ways in which extracurricular activities might shape teenagers' perspectives.

Three papers discussing the reading process make up the third section. Gao, Yang et al.'s research, “*The influence of cognitive ability in Chinese reading comprehension: can working memory updating change Chinese primary school students' reading comprehension performance?*” examines two main areas: first, evaluating the central executive (CE) for predictive effects on Chinese primary school students' reading comprehension scores; and second, investigating the impact of CE training on Chinese primary school students' reading comprehension performance. In addition, González Ramírez and Pescara Vásquez's work, “*Dimensions of reading: a study of the beliefs of language and literature preservice teachers*,” seeks to examine the attitudes of preservice language and literature teachers toward reading. To conclude this section, the study conducted by Cui et al., “*Reading for gain or reading for fun: empirical evidence from China on the adoption mechanism of integrated children's books*,” using information technology, found that Reading for Gain includes a dual effect pathway based on the Hedonic Motivation System (HMS) and the Utilitarian Motivation System (UMS).

Finally, in the fourth section, there are two books on foreign language teaching. Guo's, “*Multimodality in language education: implications of a multimodal affective perspective in foreign language teaching*,” introduces this part. It offers a multimodal affective technique to assess emotions in foreign language teaching situations. To conclude the Research Topic, Baaziz and de Vicente-Yagüe Jara's work, “*Didactics of written argumentation with Spanish as a Foreign Language (SFL) students at the university level in Algeria*,” investigates how a program that emphasizes the development of argumentation techniques has affected the quality of critical essays written by SFL students at the University of Algiers 2.

In summary, this Research Topic presents a global view that incorporates the study of critical literacy from a pluralistic research perspective. By doing so, we have provided a thorough analysis of the educational landscape that helps scholars address the issues, opportunities, and challenges that arise from the task of preparing writers and readers for a multimodal and multimedia society.

## Author contributions

ET: Conceptualization, Writing—original draft, Writing—review & editing. IS: Writing—review & editing. MJ: Writing—review & editing.

